# Association of Glycaemia Risk Index With Indices of Atherosclerosis: A Cross‐Sectional Study

**DOI:** 10.1111/1753-0407.70065

**Published:** 2025-03-06

**Authors:** Keiichi Torimoto, Yosuke Okada, Tomoya Mita, Kenichi Tanaka, Fumiya Sato, Naoto Katakami, Hidenori Yoshii, Keiko Nishida, Yoshiya Tanaka, Ryota Ishii, Masahiko Gosho, Iichiro Shimomura, Hirotaka Watada

**Affiliations:** ^1^ First Department of Internal Medicine, School of Medicine University of Occupational and Environmental Health Fukuoka Japan; ^2^ Clinical Research Center Hospital of the University of Occupational and Environmental Health Fukuoka Japan; ^3^ Department of Metabolism & Endocrinology Juntendo University Graduate School of Medicine Tokyo Japan; ^4^ Department of Metabolic Medicine Osaka University Graduate School of Medicine Osaka Japan; ^5^ Nishida Keiko Diabetes Clinic Fukuoka Japan; ^6^ Department of Biostatistics Institute of Medicine, University of Tsukuba Ibaraki Japan

**Keywords:** atherosclerosis, continuous glucose monitoring (CGM), flash glucose monitoring (FGM), glycaemia risk index (GRI), time in range (TIR), type 2 diabetes (T2DM)

## Abstract

**Aims:**

This study determined the association of the glycaemia risk index (GRI), a novel comprehensive metric derived from continuous glucose monitoring (CGM), and atherosclerosis in patients with type 2 diabetes (T2DM).

**Methods:**

We evaluated the relationship between GRI and intima‐media thickness (IMT), gray‐scale median (GSM), tissue characteristics of the carotid artery wall, and brachial‐ankle pulse wave velocity (baPWV), using baseline data from a multicenter prospective cohort study of 1000 Japanese patients with T2DM free of cardiovascular disease (CVD).

**Results:**

The study subjects were 999 patients (age: 64.6 ± 9.6 years, mean ± SD, 60.9% males, body mass index: 24.6 ± 3.9 kg/m^2^, HbA1c 7.1% ± 0.8%, TIR 78.9% ± 18.6%) with T2DM (duration of 12.9 ± 8.5 years). A higher GRI was associated with a longer duration of diabetes, a higher HbA1c level, a mean glucose level, and baPWV, and lower mean GSM. No association was noted between GRI and mean IMT. GRI was significantly associated with mean GSM (regression coefficient, *β* = −0.1277; 95% confidence interval: CI: −0.2165 to −0.0390, *p* = 0.005) and baPWV (regression coefficient, *β* = −3.1568; 95% CI: 1.5058 to 4.8079, *p* < 0.001) after adjustment for various cardiovascular risk factors.

**Conclusions:**

GRI is a potentially useful predictor of atherosclerosis in patients with T2DM. Our findings suggest that GRI, a marker of the risk of hypoglycaemia and hyperglycaemia, may serve as a clinically useful tool for the assessment of the risk of CVD in patients with T2DM, independent of the classical cardiovascular risk factors.

## Graphical abstract

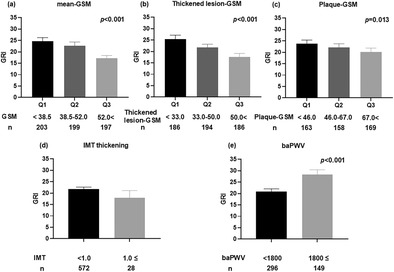

**Summary**

- The glycaemic risk index (GRI) accounts for both hypoglycaemia and hyperglycaemia, offering a comprehensive glycaemic control.
- This study described a close association between GRI and ultrasound indices of carotid artery wall tissue characteristics and atherosclerosis in patients with T2DM free of a history of cardiovascular events.
- Our findings suggest that GRI may serve as a clinically useful tool for the assessment of the risk of CVD in patients with T2DM, independent of the classical cardiovascular risk factors.

## 1 Introduction

Diabetes mellitus is currently one of the most important public health problems, with its impact continuing to increase worldwide. With a current prevalence rate of 10.5% and affecting an estimated 536.6 million individuals worldwide, the incidence of diabetes is expected to continue to increase in the future [1]. Type 2 diabetes mellitus (T2DM) is a major cause of significant health problems, including ischaemic heart disease, stroke, end‐stage renal failure, and limb amputation.

One of the pathophysiological mechanisms underlying T2DM complications is hyperglycaemia‐induced vascular endothelial damage [2] and subsequent atherosclerosis, which increases the risk of cardiovascular disease (CVD). Carotid ultrasonography is a non‐invasive procedure used to assess atherosclerotic lesions, and the gray‐scale median (GSM) value is associated with ‘vulnerable lesions’, such as carotid plaques or atheromas in the arterial wall and intraplaque [3]. Low GSM values indicate lesions rich in lipid and hemorrhagic components (not only plaque but also mean‐IMT areas), whereas high GSM values indicate lesions containing stable components such as fibrous and calcified tissue. We reported previously [4] the presence of a strong correlation between the GSM and parameters of continuous glucose monitoring (CGM) in patients with T2DM, especially time in range (TIR), mean glucose, coefficient of variation (CV), and mean amplitude of glycaemic variability (MAGE).

Recently, a new index; the glycaemic risk index (GRI), derived from the CGM data, was proposed [5] and validated by a group of 330 clinicians. GRI has been reported recently as a novel index distinct from TIR, TAR, and TBR [6]. TIR has certain limitations, such as low sensitivity to hypoglycemia and failure to describe the severity of hyperglycemia or hypoglycemia [7]. On the other hand, the novel GRI index focuses on “Very high‐glucose hyperglycemia” and “Very low‐glucose hypoglycemia”, which are strongly associated with diabetic complications and severe hypoglycemia. Thus, the use of GRI may complement the limitations of TIR. Moreover, it provides a numeric measure of glycaemic quality on a scale of 0–100, thus providing a comprehensive assessment of overall glycaemic control. The associations between the GRI and diabetes‐related complications, the diabetic retinopathy [8], and albuminuria [9] have been investigated in previous studies.

However, these studies focused primarily on microvascular complications, and only a few explored the association between the GRI and macrovascular complications. The aim of the present study was to determine the relationship between GRI and the progression of carotid atherosclerosis, including the tissue characteristics of the carotid artery wall, in patients with T2DM.

## 2 Materials and Methods

### 2.1 Study Design and Participants

This study was a sub‐analysis of data from a multicenter prospective observational cohort study [10] that examined the relationship between glycaemic variability measured using a CGM device and the incidence of cardiovascular events in Japanese patients with T2DM free of a past history of CVD. A total of 1000 patients who regularly attended outpatient clinics at 34 medical centers across Japan were recruited and followed up for up to 10 years. The baseline data from a prospective observational study were used in a cross‐sectional study to evaluate the association between GRI assessed using CGM and the ultrasonographically measured carotid lesion intima‐media thickness (IMT), GSM, and brachial‐ankle pulse wave velocity (baPWV).

The inclusion and exclusion criteria used in this study have been described in detail previously [10]. Patients aged 30–80 years with T2DM free of a history of CVD who had not changed their diabetes medications for 6 months prior to obtaining written informed consent were included. From May 2018 to March 2019, among the 1000 patients who met the eligibility criteria, 1 withdrew consent; and of the remaining 999 patients, only 600 with available baseline carotid ultrasonography imaging scans and 445 with baPWV were included in the analysis.

The study was conducted in accordance with the principles of the Declaration of Helsinki and was approved by the ethics committee of each participating medical institution. Informed consent was obtained from all participants.

### 2.2 Anthropometric and Biochemical Measurements

As described previously [10], data on diabetes history, smoking status, comorbidities, and medications used were obtained through a review of the medical records and patient interviews. Body mass index (BMI) was calculated by dividing weight (kg) by height (m^2^), and blood pressure measurements were performed using a standardized protocol. Blood samples were drawn after an overnight fast for the following laboratory tests: hemoglobin A1c (HbA1c), glucose, total cholesterol, low‐density lipoprotein (LDL) cholesterol, high‐density lipoprotein (HDL) cholesterol, triglycerides, creatinine, and uric acid, which were measured using standard methods. The estimated glomerular filtration rate (eGFR, mL/min/1.73 m^2^) was calculated according to the guidelines of the Japanese Society of Nephrology [11]. The urine albumin‐to‐creatinine ratio was measured from spot urine samples.

### 2.3 Continuous Glucose Monitoring

The FreeStyle Libre Pro (Abbott Japan, Tokyo, Japan) device was attached to each participant for 14 consecutive days, and the glucose values obtained every 15 min from days 3 to 10 were used for the analysis. The accuracy of CGM measurements is reported to be low during the first 24 h after sensor insertion (days 1–2) and the last 4 days of the 14‐day period [12]. Therefore, to ensure the reliability of the results, we limited the analysis to the FLP‐CGM data obtained from the intermediate 8‐day period. The following diurnal variability indices were obtained: CV, standard deviation (SD).

The following formula was used to calculate GRI [5]:

Percent time in

$$\text{Very}\;\mathrm{low}-\text{glucose hypoglycemia}\;\left(\text{VLow}:<54\;\mathrm{mg}/\mathrm{dL}\right)$$

$$\mathrm{Low}-\text{glucose hypoglycaemia}\;\left(\mathrm{Low}:\geq 54\;\text{to}<70\;\mathrm{mg}/\mathrm{dL}\right)$$

$$\text{High}-\text{glucose hyperglycaemia}\;\left(\text{High}:\geq 180\;\text{to}<250\;\mathrm{mg}/\mathrm{dL}\right)$$

$$\text{Very high}-\text{glucose hyperglycemia}\;\left(\text{VHigh}:\geq 250\;\mathrm{mg}/\mathrm{dL}\right)$$

$$\text{Hypoglycaemia component}\;\left(\%\right)=\text{VLow}+\left(0.8\times \mathrm{Low}\right)$$

$$\text{Hyperglycaemia component}\;\left(\%\right)=\text{VHigh}+\left(0.5\times \text{High}\right)$$

$$\begin{array}{c} \mathrm{GRI}=\left(3.0\times \text{hypoglycaemia component}\right)\\ \;+\left(1.6\times \text{hyperglycaemia component}\right) \end{array}$$

The GRI can be displayed on a two‐dimensional diagram called the GRI grid (Figure), which displays the hypocomponent on the abscissa and the hypercomponent on the ordinate. The distance from the origin represents the GRI, which is divided into five zones, from 0 to 100. These zones are color‐coded on the GRI grid in increments of 20, allowing for a visual evaluation of blood glucose quality [5].

### 2.4 IMT Measurements and Assessment of Ultrasonic Tissue Characteristics of Carotid Lesions

Ultrasound examination of the carotid arteries was performed by a professional sonographer using a high‐resolution B‐mode ultrasound scanner equipped with a high‐frequency (> 7.5 MHz) linear transducer with a detection limit of < 0.1 mm [10]. The common carotid artery (CCA), carotid bulb, and internal carotid artery were scanned in transverse and longitudinal sections at different angles.

IMT was defined as the distance between two parallel echo lines corresponding to the vessel lumen and adventitia. To avoid inter‐reader variability, all scans were stored electronically and sent to the central office (IMT Evaluation Committee, Osaka, Japan). The scans were analyzed using an automated digital edge detection software (IntimaScope; Media Cross, Tokyo) and read in random order by a single experienced reader blinded to the clinical characteristics of the patients [13]. The software system averaged approximately 200 IMT values at a segment 2 cm proximal to the carotid valve dilatation in the left and right CCAs, with the mean value of the left and right CCAs defined as the mean IMT. The maximum IMT of the left and right CCAs, including plaque lesions, was also measured, and the maximum values of the left and right CCAs were defined as the CCA‐max‐IMT. In this study, ‘carotid plaque’ was defined as a localized raised lesion with an inflection point on the surface of the intima‐media complex and a maximum thickness ≥ 1.0 mm.

Results of the carotid lesion ultrasound were evaluated using the GSM. Image standardization and grayscale values were calculated using Adobe Photoshop version 7.0 (Adobe Systems, San Jose, CA, USA). B‐mode images were standardized using the curve option to obtain the GSM values of 0–5 for blood and 185–195 for the outer membrane [14]. The left and right mean IMT regions (segments located 2 cm proximal to the dilatation of the carotid valve) were drawn using the freehand tool, and the GSM values of the selected regions were obtained from the entire drawn region. The average value of each of the left and right carotid arteries was defined as the ‘Mean‐GSM’.

If atheromatous plaque lesions and/or thickened lesions (local IMT ≥ 1.0 mm) were detected, the GSM of these lesions was also measured in the same manner. The lowest value of these lesions was defined as ‘Thickened lesion‐GSM’. When multiple plaque lesions were detected in the same individual, the GSM of the thickest plaque was measured in the left and right carotid arteries separately, and the lower value, termed ‘plaque‐GSM’, was then used as the representative value of the plaque in the participant.

To avoid inter‐reader variability, all scans were read in a random order by a single reader who was unaware of the clinical characteristics of the patients. The intra‐reader variability of GSM measurements was 2.9% across 40 consecutive measurements.

### 2.5 Measurement of Brachial‐Ankle Pulse Wave Velocity (baPWV)

The baseline baPWV was measured using an automatic waveform analyzer (BP‐203RPE form; Colin Medical Technology, Komaki, Japan) [15]. Briefly, the measurements were performed with the patient in a supine position after a 5‐min bed rest. Occlusion and monitoring cuffs were securely applied to the arms and ankles, and pressure waveforms were simultaneously recorded from the brachial artery using an oscillometric method.

All scans were performed by trained observers at each site. Patients with an ankle‐brachial pressure ratio < 0.90 were considered to have peripheral arterial disease, and their baPWV data were excluded from the study. Based on this criterion, data from 1 of the 446 patients who underwent baPWV evaluation were excluded.

### 2.6 Statistical Analysis

Data were expressed as means and standard deviations or medians and interquartile ranges for continuous variables and as counts and percentages for categorical variables. First, the overall patient background was compared according to the GRI tertiles (*n* = 999). Further, IMT, GSM, and baPWV were compared according to the GRI tertiles in 602 patients with available baseline carotid ultrasound imaging scans and 445 patients with available baPWV data. In addition, multivariate regression analysis was performed to determine the association of GRI with IMT, GSM, and baPWV. Model 1 remained unadjusted, whereas Model 2 was adjusted for conventional major risk factors for CVD by including age and sex, BMI, diabetes duration, HbA1c, systolic blood pressure, total cholesterol, HDL cholesterol, log‐transformed triglycerides, eGFR, uric acid, log‐transformed urine albumin‐to‐creatinine ratio, smoking status, alcohol consumption, insulin therapy, use of angiotensin‐converting enzyme (ACE) inhibitors and/or angiotensin II receptor blockers (ARBs), statins, antiplatelet medications, and the presence of retinopathy. Model 3 was defined as Model 2 with LDL cholesterol replacing total cholesterol. A *p* value < 0.05 was considered significant. All analyses were performed using SAS software (version 9.4, SAS Institute, Cary, NC, USA).

## 3 Results

### 3.1 Clinical Characteristics of Study Population

Table 1, Tables S1 and S2 summarize the baseline clinical characteristics of the participants (*n* = 999). The mean age of the participants was 64.6 ± 9.6 years, with 60.9% being males. The HbA1c level was 7.1% ± 0.8% (53.5 ± 9.0 mmol/mol), whereas the estimated duration of T2DM was 12.9 ± 8.5 years. The mean GRI was 23.6 ± 22.7, with the tertile ranges as follows: Q1 (Low GRI, < 11.15), Q2 (Middle GRI, ≥ 11.15 to < 25.39), and Q3 (High GRI, ≥ 25.39). The mean GRI of the tertile ranges was 5.8 ± 3.0 (Low GRI), 17.0 ± 4.1 (Middle GRI), and 48.1 ± 23.7 (High GRI). The GRI grid for each range is shown in Figure S1. The High GRI group had a longer duration of diabetes, higher HbA1c level, mean glucose level, CV, TAR^> 180 mg/dL^, TAR^> 250 mg/dL^, TBR^< 70 mg/dL^, TBR^< 54 mg/dL^, MODD, IQR, and baPWV, while TIR, Mean‐GSM, Thickened lesion‐GSM, and Plaque‐GSM were low. The proportions of patients who received sulfonylurea and insulin therapy and developed neuropathy, retinopathy, and nephropathy were high in the High GRI group (Table 1 and Table S2).

**TABLE 1:** Clinical characteristics of study participants.

Variables	All	Low GRI (< 11.15)	Middle GRI (≥ 11.15 to < 25.39)	High GRI (≥ 25.39)	*p* value
*n*	999	335	330	334	
Age, years	64.6 ± 9.6	65.4 ± 9.1	65.4 ± 9.3	64.7 ± 10.4	0.005
Gender, males/females	608/391	202/133	204/126	202/132	0.914^a^
Duration of diabetes, years	12.9 ± 8.5	10.8 ± 7.9	13.5 ± 8.8	14.3 ± 8.4	< 0.001
Body mass index, kg/m^2^	24.6 ± 3.9	24.9 ± 3.9	24.4 ± 3.7	24.5 ± 4.0	0.202
Systolic blood pressure, mmHg	131.2 ± 14.8	130.7 ± 14.1	131.4 ± 14.4	131.6 ± 15.9	0.892
Diastolic blood pressure, mmHg	75.5 ± 11.0	76.3 ± 10.7	75.4 ± 10.9	75.0 ± 11.6	0.165
HbA1c, %	7.1 ± 0.8	6.6 ± 0.5	7.0 ± 0.6	7.6 ± 0.8	< 0.001
Total cholesterol, mg/dL	185.8 ± 31.6	187.5 ± 31.0	184 ± 30.4	185.8 ± 185.8	0.365
LDL cholesterol, mg/dL	103.1 ± 26.5	105.1 ± 26.1	102 ± 25	102.1 ± 26.5	0.280
HDL cholesterol, mg/dL	60.4 ± 15.7	60.3 ± 15.8	60.9 ± 15.2	60 ± 15.7	0.774
Triglycerides, mg/dL	99.0 [71.0, 140.0]	96.0 [71.0, 136.0]	98.0 [71.0, 141.0]	103.0 [72.0, 143.0]	0.246
Uric acid, mg/dL	5.17 ± 1.23	5.26 ± 1.21	5.13 ± 1.2	5.12 ± 1.23	0.188
eGFR, mL/min/1.73 m^2^	73.4 ± 20.6	73.4 ± 17.6	74.5 ± 19.5	72.3 ± 24.3	0.131
u‐Alb/Cr, mg/g · Cre	14.4 [6.4, 44.5]	10.0 [5.3, 28.4]	14.0 [7.0, 38.2]	22.3 [8.0, 75.6]	< 0.001
Neuropathy, *n* (%)	286 (28.6)	73 (21.8)	82 (24.8)	131 (39.2)	< 0.001^a^
Retinopathy, *n* (%)	222 (22.2)	54 (16.1)	65 (19.7)	103 (30.8)	< 0.001^a^
Nephropathy, *n* (%)	203 (20.3)	42 (12.5)	67 (20.3)	94 (28.1)	< 0.001^a^
Use of oral glucose‐lowering agents, *n* (%)					
Metformin	543 (54.4)	178 (53.1)	187 (56.7)	178 (53.3)	0.589^a^
Sulfonylurea	127 (12.7)	21 (6.3)	43 (13.0)	63 (18.9)	< 0.001^a^
Glinide	68 (6.8)	13 (3.9)	22 (6.7)	33 (9.9)	0.008^a^
Dipeptidyl peptidase‐4 inhibitors	577 (57.8)	194 (57.9)	186 (56.4)	197 (59.0)	0.789^a^
Sodium‐glucose cotransporter‐2 inhibitors	231 (23.1)	66 (19.7)	78 (23.6)	87 (26.0)	0.145^a^
Thiazolidinediones	143 (14.3)	35 (10.4)	50 (15.2)	58 (17.4)	0.030^a^
α‐glucosidase inhibitor	172 (17.2)	77 (23.0)	48 (14.5)	47 (14.1)	0.004^a^
Glucagon‐like peptide‐1 antagonists	74 (7.4)	13 (3.9)	21 (6.4)	40 (12.0)	< 0.001^a^
Insulin	158 (15.8)	11 (3.3)	50 (15.2)	97 (29.0)	< 0.001^a^
Use of antihypertensive drugs, *n* (%)	483 (48.3)	160 (47.8)	156 (47.3)	167 (50.0)	0.752^a^
ACE inhibitors	28 (2.8)	8 (2.4)	9 (2.7)	11 (3.3)	0.766^a^
Angiotensin II receptor blockers	390 (39.0)	133 (39.7)	127 (38.5)	130 (38.9)	0.949^a^
Calcium channel blockers	273 (27.3)	84 (25.1)	95 (28.8)	94 (28.1)	0.512^a^
Use of lipid‐lowering agents, *n* (%)	595 (59.7)	207 (61.8)	196 (59.8)	192 (57.5)	0.531^a^
Statins	508 (51.0)	172 (51.3)	170 (51.8)	166 (49.7)	0.850^a^
Ezetimibe	107 (10.7)	45 (13.4)	36 (11.0)	26 (7.8)	0.060^a^
Fibrates	41 (4.1)	14 (4.2)	10 (3.0)	17 (5.1)	0.060^a^
Use of antithrombotic agents, *n* (%)	64 (6.4)	22 (6.6)	21 (6.4)	21 (6.3)	1.000^a^
Antiplatelet agents	50 (5.0)	16 (4.8)	16 (4.8)	18 (5.4)	0.934^a^
Anticoagulants	15 (1.5)	7 (2.1)	5 (1.5)	3 (0.9)	0.445^a^
FLP‐CGM‐derived metrics					
Mean glucose, mg/dL	140.5 ± 32.3	122.1 ± 11.1	134 ± 16.9	165.2 ± 41.6	< 0.001
SD, mg/dL	36.7 ± 11.3	27.7 ± 5.2	36.1 ± 6.2	46.4 ± 12	< 0.001
CV, %	26.2 ± 5.8	22.7 ± 3.8	27.1 ± 4.6	28.8 ± 6.7	< 0.001
TIR, %	78.9 ± 18.6	94.1 ± 3.6	83.4 ± 5.8	59.2 ± 18.6	< 0.001
TAR^> 180 mg/dL^, %	19 ± 19.2	5.3 ± 3.8	14.8 ± 7.7	36.8 ± 22.5	< 0.001
TAR^> 250 mg/dL^, %	3.8 ± 9.3	0.1 ± 0.3	0.9 ± 1.1	10.5 ± 13.9	< 0.001
TBR^< 70 mg/dL^, %	2.2 ± 4.7	0.6 ± 0.9	1.8 ± 2.5	4.1 ± 7.3	0.033
TBR^< 54 mg/dL^, %	0.3 ± 1.5	0 ± 0.1	0.1 ± 0.3	0.8 ± 2.5	< 0.001
GRI	16.4 [8.3, 31.3]	5.8 [3.4, 8.3]	16.4 [13.3, 20.0]	39.8 [31.1, 58.1]	< 0.001
Hypoglycaemia component of GRI	0.1 [0.0, 1.6]	0.2 [0.0, 0.8]	0.3 [0.0, 2.5]	0.0 [0.0, 4.8]	0.033
Hyperglycemia component of GRI	6.7 [2.3, 15.7]	2.3 [1.2, 4.2]	8.0 [5.1, 11.0]	20.6 [13.5, 30.1]	< 0.001
Ultrasonographic scans of the artery, n	602	224	204	172	
Mean‐IMT, mm	0.76 ± 0.15	0.75 ± 0.17	0.77 ± 0.15	0.76 ± 0.13	0.169
CCA‐max, mm	1.11 ± 0.44	1.08 ± 0.41	1.12 ± 0.46	1.13 ± 0.46	0.096
Mean‐GSM	48.7 ± 19.3	52.5 ± 21.2	47.6 ± 17.4	44.9 ± 17.9	< 0.001
Thickened lesion‐GSM	43.5 ± 19.5	47.7 ± 21.5	42.1 ± 18.6	39.6 ± 16.7	< 0.001
Plaque‐GSM	61.5 ± 29.9	66.6 ± 32.6	58.3 ± 25.6	59.1 ± 30.5	0.031
Arterial stiffness, *n*	445	169	134	142	
PWV, cm/s	1706 ± 367	1623 ± 306	1697 ± 313	1813 ± 449	< 0.001

*Note:* Data are mean ± SD or *n* (%), median [interquartile range]. *p* values represent differences among the Low, Middle, and High GRI groups, assessed by the Kruskal–Wallis test.

Abbreviations: baPWV, brachial‐ankle pulse wave velocity; CCA, common carotid artery; CV, coefficient of variation; GRI, glycemia risk index; GSM, gray scale median; HbA1c, hemoglobin A1c; HBGI, high blood glucose index; IMT, intima media thickness; IQR, interquartile range; LBGI, low blood glucose index; MODD, means of daily differences; SD, standard deviation; TAR, time above range; TBR, time below range; TIR, time in range.

^a^ χ^2^ test was used to determine the association between differences among the Low, Middle, and High GRI groups.

### 3.2 Associations of Carotid Measures With GRI

**FIGURE 1:**
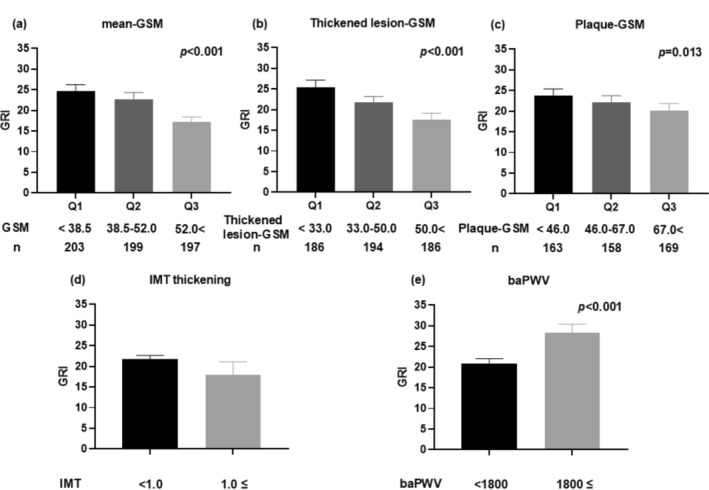
Relationship between GRI and indices of arteriosclerosis. (a) GRI in tertiles (Q1–Q3) of GSM. Q1: < 38.5 (*n* = 203), Q2: ≥ 38.5 to < 52.0 (*n* = 199), Q3: ≥ 52.0 (*n* = 197). (b) The GRI in tertiles (Q1–Q3) of thickened lesion‐GSM. Q1: < 33.0 (*n* = 186), Q2: ≥ 33.0 to < 50.0 (*n* = 194), Q3: ≥ 50.0 (*n* = 186). (c) The GRI in tertiles (Q1–Q3) of plaque‐GSM. Q1: < 46.0 (*n* = 163), Q2: ≥ 46.0 to < 67.0 (*n* = 158), Q3: ≥ 67.0 (*n* = 169). (d) The GRI in groups with [IMT‐thickness: IMT ≥ 1.0 mm (*n* = 28)], and without IMT‐thickening [IMT‐thickness: IMT < 1.0 mm (*n* = 572)]. (e) GRI of the high [baPWV ≥ 1800 cm/s (*n* = 149)] and low [baPWV < 1800 cm/s (*n* = 296)] arterial stiffness groups. baPWV, brachial‐ankle pulse wave velocity; GRI, glycaemia risk index; GSM, grayscale median; IMT, intima‐media thickness.

The mean IMT for the entire group was 0.76 ± 0.15 mm. Of the 602 patients, 28 exhibited IMT thickening (mean IMT ≥ 1.0 mm), with no significant difference in GRI between those with IMT thickening (mean IMT ≥ 1.0 mm, *n* = 28) and those without IMT thickening (mean IMT < 1.0 mm, *n* = 572) (Figure 1).

The Mean‐GSM for the entire group was 48.7 ± 19.3. Comparison of the GRI between the GSM tertile groups showed a higher GRI in the group with the lowest Mean‐GSM (Figure 1). The Thickened lesion‐GSM was 43.5 ± 19.3. The GRI was higher in the group with a lesser Thickened lesion‐GSM (Figure 1). The Plaque‐GSM was 61.5 ± 29.9. Comparison of the GRI between Plaque‐GSM tertiles showed a higher GRI in the group with a lower Plaque‐GSM (Figure 1).

Table 2 presents the results of the linear regression analysis examining the association of GRI with IMT and GSM. GRI was not significantly associated with the mean IMT. In contrast, GRI was associated with the Mean‐GSM (Model 1, Table 2). In addition, it was significantly associated with GSM (regression coefficient, *β* = −0.1392; 95% CI: −0.2323 to −0.0460, *p* = 0.003) after adjustment for cardiovascular risk factors, such as age, sex, BMI, duration of diabetes, smoking status, alcohol consumption, insulin therapy, use of ACE inhibitors and/or ARBs, statins, antiplatelet medications, presence of retinopathy, systolic blood pressure, eGFR, HbA1c, total cholesterol, HDL cholesterol, log‐transformed triglyceride, and uric acid levels, and log‐transformed urine albumin‐creatinine ratio (Model 2, Table 2). The model in which total cholesterol was replaced with LDL cholesterol yielded similar results (Model 3, Table S3).

**TABLE 2:** Association of GRI with intima‐media thickness and gray‐scale median.

		Mean‐IMT (*n* = 600)		Mean‐GSM (*n* = 599)	
		*β* (95% CI)	*p* value	*β* (95% CI)	*p* value
**Model 1**					
GRI		0.0002 (−0.0004, 0.0008)	0.458	−0.1485 (−0.2242, −0.0728)	< 0.001
**Model 2**					
GRI		0.0003 (−0.0004, 0.0010)	0.403	−0.1392 (−0.2323, −0.0460)	0.003
Age		0.0045 (0.0030, 0.0060)	< 0.001	−0.3419 (−0.5377, −0.1462)	< 0.001
Gender, female		−0.0348 (−0.0659, −0.0036)	0.029	−2.0016 (−5.9472, 1.9441)	0.319
BMI		−0.0023 (−0.0059, 0.0014)	0.221	−1.0672 (−1.5285, −0.6060)	< 0.001
Duration of diabetes		−0.0001 (−0.0018, 0.0015)	0.889	−0.3173 (−0.5285, −0.1062)	0.003
HbA1c		−0.0111 (−0.0297, 0.0076)	0.244	0.9018 (−1.4561, 3.2596)	0.453
Systolic blood pressure		0.0015 (0.0007, 0.0023)	< 0.001	0.0796 (−0.0251, 0.1842)	0.136
Total cholesterol		0.0049 (−0.014, 0.0238)	0.609	0.1316 (−2.2609, 2.5241)	0.914
HDL cholesterol		−0.025 (−0.0624, 0.0123)	0.189	3.6313 (−1.0984, 8.3610)	0.132
Log‐transformed triglycerides		−0.0074 (−0.036, 0.0211)	0.610	−4.2911 (−7.9076, −0.6747)	0.020
eGFR		−0.0003 (−0.0011, 0.0005)	0.502	−0.1232 (−0.2233, −0.0232)	0.016
Uric acid		0.000 (−0.0002, 0.0002)	0.749	0.0067 (−0.0179, 0.0314)	0.593
Log‐transformed u‐Alb		0.0014 (−0.0073, 0.0100)	0.759	−0.2734 (−1.3747, 0.8278)	0.626
Smoking	Never	Reference	0.185	Reference	0.166
	Current	0.0180 (−0.0172, 0.0532)		4.3042 (−0.1536, 8.7620)	
	Former	0.0277 (−0.0020, 0.0574)		1.8147 (−1.9518, 5.5812)	
Alcohol consumption		−0.0204 (−0.0461, 0.0053)	0.120	−0.5695 (−3.8221, 2.6831)	0.731
Use of insulin therapy		−0.0146 (−0.0504, 0.0213)	0.426	−4.1084 (−8.6752, 0.4585)	0.078
Use of ACE‐i and/or ARB		0.0081 (−0.0181, 0.0344)	0.543	5.3518 (2.0247, 8.6789)	0.002
Use of statin		0.0084 (−0.0179, 0.0346)	0.531	−5.513 (−8.8371, −2.1890)	0.001
Use of antiplatelet agents		0.0162 (−0.0430, 0.0754)	0.591	−5.1762 (−12.6717, 2.3193)	0.175
Presence of retinopathy		0.0203 (−0.0098, 0.0503)	0.186	3.1164 (−0.6948, 6.9276)	0.109

*Note:* Data are results of univariable and multivariable linear regression analysis.

Abbreviations: ACE, angiotensin‐converting enzyme; ACE‐i and/or ARB, ACE inhibitor and/or Angiotensin II receptor blockers; BMI, body mass index; CI, confidence interval; eGFR, estimated glomerular filtration rate; GRI, gray‐scale median; HbA1c, hemoglobin A1c; HbA1c, hemoglobin A1c; HDL, high‐density lipoprotein; log‐transformed u‐Alb, log‐transformed urine albumin‐to‐creatinine ratio.

The relationships between thickened‐GSM or plaque‐GSM and GRI, its hyperglycemia component, and its hypoglycemia component, as well as the relationships between mean‐IMT or mean‐GSM and the hyperglycemia or hypoglycemia component of GRI, are shown in Tables S5–S9.

### 3.3 Association of baPWV With GRI

The mean baPWV of the entire group (*n* = 445) was 1706 ± 367 cm/s. GRI was significantly higher in the high baPWV group (mean baPWV ≥ 1800, *n* = 149) than in the low baPWV group (mean baPWV < 1800, *n* = 296) (Figure 1). Table 3 presents the results of the linear regression analysis that examined the relationship of GRI with baPWV. GRI was significantly associated with baPWV (Model 1, Table 3). Furthermore, GRI was significantly associated with baPWV (regression coefficient, *β* = 3.1692; 95% CI: 1.4085 to 4.9299, *p* < 0.001) after adjustment for cardiovascular risk factors, such as age, sex, BMI, duration of diabetes, smoking status, alcohol consumption, insulin therapy, use of ACE inhibitors and/or ARBs, statins, antiplatelet medications, the presence of retinopathy, systolic blood pressure, eGFR, HbA1c, total cholesterol, HDL cholesterol, log‐transformed triglyceride, uric acid, and log‐transformed urine albumin levels (Model 2, Table 3). The model in which total cholesterol was replaced with LDL cholesterol yielded similar results (Model 3, Table S4).

**TABLE 3:** Association of GRI with baPWV.

		Mean‐baPWV (*n* = 445)	
		*β* (95% CI)	*p* value
**Model 1**			
GRI		3.5628 (2.0579, 5.0677)	< 0.001
**Model 2**			
GRI		3.1692 (1.4085, 4.9299)	< 0.001
Age		13.8791 (10.0088, 17.7495)	< 0.001
Gender, female		55.2767 (−20.9861, 131.5395)	0.155
BMI		−14.1964 (−23.2772, −5.1156)	0.002
Duration of diabetes		4.0549 (0.1958, 7.9140)	0.040
HbA1c		−67.5610 (−117.0135, −18.1085)	0.008
Systolic blood pressure		6.4153 (4.4140, 8.4165)	< 0.001
Total cholesterol		−43.0021 (−90.6241, 4.6198)	0.077
HDL cholesterol		−1.2344 (−92.7286, 90.2599)	0.979
Log‐transformed triglycerides		73.9262 (7.6859, 140.1665)	0.029
eGFR		0.4609 (−1.3576, 2.2795)	0.619
Uric acid		0.0995 (−0.3644, 0.5634)	0.673
Log‐transformed u‐Alb		42.7150 (21.6544, 63.7756)	< 0.001
Smoking	Never	Reference	0.377
	Current	−22.6465 (−107.4307, 62.1377)	
	Former	32.4630 (−39.0861, 104.0121)	
Alcohol consumption		38.9937 (−23.7578, 101.7452)	0.223
Use of insulin therapy		57.0183 (−32.2703, 146.3070)	0.210
Use of ACE‐i and/or ARB		−14.6314 (−78.7153, 49.4525)	0.654
Use of statin		−70.2920 (−133.4258, −7.1582)	0.029
Use of antiplatelet agents		12.7553 (−126.8826, 152.3932)	0.858
Presence of retinopathy		15.4775 (−56.2721, 87.2270)	0.672

*Note:* Results of univariable and multivariable linear regression analysis. See Table 2 for abbreviations.

The relationships between baPWV and the hyperglycemia or hypoglycemia component of GRI are shown in Tables S10 and S11.

## 4 Discussion

The present study described a close association between GRI and ultrasound indices of carotid artery wall tissue characteristics and atherosclerosis, as assessed by PWV, in patients with T2DM free of a history of cardiovascular events (Figure S2). Our study is the first to report that patients with high GRI, a novel composite CGM index, also have a low Mean‐GSM, which reflects changes in the histopathological properties of the vessel wall. This relationship remained significant even after adjustment for other cardiovascular risk factors and HbA1c level, suggesting that GRI is related to carotid artery wall histopathological properties independent of the presence or absence of chronic hyperglycaemia and major cardiovascular risk factors.

GRI is a single‐number summary of the risk of hypoglycaemia and hyperglycaemia, based on the opinion of experienced clinicians [5]; its usefulness has been reported in the Asia‐Pacific consensus recommendations for the application of CGM [9, 16, 17]. Previous studies explored the relationship between GRI and long‐term diabetic microvascular complications. A cohort study of 1204 adults with T2DM concluded that a higher GRI was associated with a higher risk of developing diabetic retinopathy [8]. Another study showed that GRI was strongly associated with albuminuria and was an independent risk factor for macroalbuminuria [17]. In the present study, significantly higher incidence rates of neuropathy, retinopathy, nephropathy, and albuminuria were noted in the High GRI group. This finding indicates a close association between GRI and diabetic microvascular disease. Considering the relationship with atherosclerotic disease, the results of the present study are in agreement with the results of a recent study of 342 patients with T2DM that showed the association of this novel CGM index with baPWV [18]. Our study is the first to demonstrate the association of Mean‐GSM, which reflects histopathological changes related to atheromatous plaque and intra‐plaque hemorrhage, with GRI.

GRI is a novel index that focuses on “Very high‐glucose hyperglycemia” and “Very low‐glucose hypoglycemia”, which are strongly associated with diabetic complications and severe hypoglycemia [7]. However, one of the main limitations of GRI is that it becomes less useful as an index of glycemic control in patients with well‐controlled diabetes, where TIR approaches 100%. This is because GRI depends heavily on fluctuations in blood glucose levels. Thus, GRI may not be suitable for assessing glycemic control in patients with early‐stage type 2 diabetes or impaired glucose tolerance. In comparison, GRI is more useful in populations with significant glucose fluctuations, such as those with postprandial hyperglycemia and hypoglycemia, two factors known to increase the risk of progression of atherosclerosis.

A recent study using GSM showed that ultrasound histopathological characterization of the carotid artery improved the risk prediction of cardiovascular events in patients with asymptomatic T2DM suffering carotid plaques [19]. Furthermore, a study of 262 patients who underwent carotid endarterectomy showed that a low GSM was a risk factor for cardiovascular events [20]. Several previous studies, including those conducted by our group [4, 21, 22, 23], also documented the presence of a close association between tissue characteristics of coronary artery plaques and indices of glycaemic variability, such as MAGE, SD, MODD, and TIR. In the present study, a robust relationship was observed between the tissue characteristics of coronary artery plaques and GRI.

Ample evidence suggests that the early stages of atherosclerogenesis correlate with vascular endothelial dysfunction [24]. Furthermore, blood glucose fluctuations are known to negatively affect vascular endothelial function in patients with normal glucose levels [25] and T2DM [26, 27], and oxidative stress [28] caused by fluctuations in blood glucose levels is thought to have detrimental effects. Intermittent hyperglycaemia increases oxidative stress‐related cell apoptosis in vascular endothelial cells through PKC‐dependent activation of NAD(P)H oxidase [29] and the expression of several adhesion molecules, such as ICAM‐1, VCAM‐1, and E‐selectin [30]. Hyperglycaemia also inhibits NO production in arterial vascular endothelial cells and stimulates the production of plasminogen activator inhibitor‐1 [31].

The relationship between hypoglycaemia, hyperglycaemia, and cardiovascular events is an important pathophysiological factor in the development of cardiovascular complications [32]. High levels of catecholamines [33] and decreased sensitivity to prostacyclin [34] that occur during hypoglycaemia lead to platelet activation and play important roles in the initiation and progression of the atherosclerogenesis process [35]. Furthermore, the effects of hypoglycaemia on the induction and increased levels of inflammatory markers, such as interleukin (IL)‐6 and IL‐8, act to worsen endothelial damage and coagulation abnormalities and increase the risk of CVD events. Indeed, a previous study involving 27 patients with T2DM reported that hypoglycaemia induces vascular endothelial dysfunction [36]. The GRI is an index weighted according to the risk of hypoglycaemia, particularly severe hypoglycaemia, and may allow risk assessment of atherosclerotic disease from a hypoglycaemic perspective, unlike TIR and CGM indices of glycaemic variability.

Our results showed no significant relationship between GRI and IMT. The association between IMT and glycaemic variability remains debatable; several previous studies on T2DM reported the lack of an IMT‐glycaemic variability association [37, 38]. In contrast, several studies have reported significant associations between IMT and measures of glycaemic variability, such as MAGE and SD [39, 40]. A large cross‐sectional study of 2215 patients with T2DM found no association between IMT and measures of glycaemic variability, such as MAGE and SD, after adjusting for classical cardiovascular risk factors [41].

The strength of this study is its multicenter design, using data from a prospective cohort to assess cardiovascular events. This study design enabled the evaluation of the association between GRI and ultrasound tissue characteristics of the carotid artery wall and baPWV, independent of various cardiovascular risk factors. However, this study has some limitations. First, although the study found a close association between GRI, a parameter derived from CGM, and ultrasound tissue characteristics of the carotid artery wall and baPWV, it remains unclear whether this association is causal, given the cross‐sectional nature of the study. For this reason, we have embarked on a long‐term follow‐up study [10] in the same cohort. Second, this study included a relatively small sample size, and all subjects were Japanese. Therefore, future validation in a larger multi‐ethnic cohort is warranted. Third, the effects of potential confounders could not be excluded. Whereas the data analysis provided control for confounders, such as sex and smoking status, in the multivariate regression analysis, this could not include potential risk factors for atherosclerosis, such as insulin resistance [42, 43]. Fourth, our study included only a few cases with hypoglycemia, with 23% of GRI attributed to the hypoglycemia component and 77% to the hyperglycemia component. Our results need to be confirmed in future studies involving populations with higher percentages of patients with hypoglycemia.

## 5 Conclusions

In conclusion, this study showed that GRI, a novel composite CGM index of hypo‐ and hyperglycemia, is associated with ultrasound tissue characteristics of the carotid artery wall and arterial stiffness in patients with T2DM free of cardiovascular events. This finding suggests that GRI can potentially be used for the prediction of cardiovascular disease development in such patients. However, large prospective studies are warranted to confirm the association of GRI with arterial wall stiffness and other macrovascular complications.

## Author Contributions

All authors contributed to the study design and collection of clinical data. K.T. drafted the manuscript. M.G., the biostatistician, coordinated and supervised data analysis. K.T., Y.O., T.M., K.T., F.S., N.K., H.Y., K.N., Y.T., R.I., M.G., I.S., and H.W. collected, analysed, and interpreted the data; reviewed and edited the manuscript; and approved the final version of the manuscript. Y.O. and H.W. are the principal guarantors of this work; they have full access to all study data and take responsibility for the integrity of the data and the accuracy of data analysis. All authors have read and agreed to the publication of this manuscript.

## Conflicts of Interest

The authors declare no conflicts of interest.

## Supporting information


**Data S1.** Supporting Information.
**Figure S1.** The Glycemia risk index grid. The Glycemia risk index (GRI) is displayed on a two‐dimensional diagram called the GRI grid, which displays the hypocomponent on the abscissa and the hypercomponent on the ordinate. The distance from the origin represents the overall GRI score, which is divided into five zones (A–E), from 0 to 100. These zones are color‐coded on the GRI grid in increments of 20, allowing for a visual evaluation of blood glucose quality. GRI is analyzed in tertiles (Q1–Q3) as follows: Q1 (Low GRI, < 11.15), Q2 (Middle GRI, ≥ 11.15 to < 25.39), and Q3 (High GRI, ≥ 25.39).
**Figure S2.** Summary of this study. This study demonstrates a close association between GRI and ultrasound indices of carotid artery wall tissue characteristics (GSM) and atherosclerosis, as assessed by PWV, in patients with T2DM free of history of cardiovascular events. GRI, glycaemia risk index; GSM, grayscale median; PWV, pulse wave velocity; TRI, Time in range.
