# Correction to “Association of Glycaemia Risk Index With Indices of Atherosclerosis: A Cross‐Sectional Study”

**DOI:** 10.1111/1753-0407.70092

**Published:** 2025-04-25

K. Torimoto, Y. Okada, T. Mita, et al., “Association of Glycaemia Risk Index With Indices of Atherosclerosis: A Cross‐Sectional Study,” *Journal of Diabetes* 17, no. 3 (2025): e70065, https://doi.org/10.1111/1753‐0407.70065.

In Results of the “ABSTRACT” section, the text “GRI was significantly associated with mean GSM (regression coefficient, *β* = −0.1277; 95% confidence interval: CI: −0.2165 to −0.0390, *p* = 0.005) and baPWV (regression coefficient, *β* = −3.1568; 95% CI: 1.5058 to 4.8079, *p* < 0.001) after adjustment for various cardiovascular risk factors.” was incorrect. This should have read: “GRI was significantly associated with mean GSM (regression coefficient, *β* = −0.1392; 95% confidence interval: CI: −0.2323 to −0.0460, *p* = 0.003) and baPWV (regression coefficient, *β* = 3.1692; 95% CI: 1.4085 to 4.9299, *p* < 0.001) after adjustment for various cardiovascular risk factors.”

We apologize for this error.

