# Supplementary material for: Association of Glycaemia Risk Index With Indices of Atherosclerosis: A Cross‐Sectional Study
Source: J Diabetes. 2025 Mar 6;17(3):e70065. doi: 10.1111/1753-0407.70065 (PMC11884825; doi:10.1111/1753-0407.70065)

Table S1. Clinical characteristics of study participants who underwent carotid ultrasonography or brachial-ankle pulse wave velocity (baPWV)

Variables	carotid ultrasonography	baPWV
n	602	445
Age, years	64.9±9.2	65.9±9.0
Gender, males/females	380/220	301/144
Duration of diabetes, years	13.3±8.3	13.5±8.3
Body mass index, kg/m^2	24.6±3.8	24.4±3.7
Systolic blood pressure, mmHg	132.0±14.8	132.5±14.8
Diastolic blood pressure, mmHg	76.4±10.9	77.1±11.2
HbA1c, %	7.0±0.8	7.1±0.8
Total cholesterol, mg/dL	184.4±31.2	190.1±30.0
LDL cholesterol, mg/dL	102.5±25.9	107.0±24.6
HDL cholesterol, mg/dL	59.9±15.8	60.1±15.6
Triglycerides, mg/dL	98.0 [72.0, 138.0]	99.0 [71.0, 140.0]
Uric acid, mg/dL	5.19±1.23	5.22±1.24
eGFR, mL/min/1.73 m^2	70.0±18.2	70.8±18.3
u-Alb/Cr, mg/g・Cre	13.7 [6.0, 41.0]	13.9 [6.3, 40.9]
Neuropathy, n (%)	206 (34.2)	152 (34.2)
Retinopathy, n (%)	147 (24.4)	108 (24.3)
Nephropathy, n (%)	149 (24.8)	123 (27.6)
Use of oral glucose-lowering agents, n (%)		
Metformin	339 (56.3)	234 (52.6)
Sulfonylurea	69 (11.5)	59 (13.3)
Glinide	38 (6.3)	25 (5.6)
Dipeptidyl peptidase-4 inhibitors	334 (57.1)	252 (56.6)
Sodium-glucose cotransporter-2 inhibitors	149 (24.8)	102 (22.9)
Thiazolidinediones	94 (15.6)	55 (12.4)
α-glucosidase inhibitor	131 (21.8)	124 (27.9)
Glucagon-like peptide-1 antagonists	42 (7.0)	20 (4.5)
Insulin	97 (16.1)	63 (14.2)
Use of antihypertensive drugs, n (%)	309 (51.3)	233 (52.4)
ACE inhibitors	12 (2.0)	18 (4.0)
Angiotensin II receptor blockers	256 (42.5)	192 (43.1)
Calcium channel blockers	165 (27.4)	117 (26.3)
Use of lipid-lowering agents, n (%)	376 (62.7)	254 (57.1)
Statins	320 (53.3)	207 (46.5)
Ezetimibe	74 (12.3)	43 (9.7)
Fibrates	24 (4.0)	20 (4.5)
Use of antithrombotic agents, n (%)	33 (5.5)	24 (5.4)
Antiplatelet agents	25 (4.2)	19 (4.3)
Anticoagulants	9 (1.5)	5 (1.1)
FLP-CGM-derived metrics		
Mean glucose, mg/dL	137.3±30.3	142.0±32.0
SD, mg/dL	35.3±10.8	36.5±11.8
CV, %	25.8±5.9	25.6±5.8
TIR, %	80.9±17.7	78.7±19.8
$TAR^{>180mg/dL}$, %	17.0±18.2	19.6±20.1
$TAR^{>250mg/dL}$, %	3.1±8.0	4.0±9.1
$TBR^{<70mg/dL}$, %	2.2±4.7	1.8±4.3
$TBR^{<54mg/dL}$, %	0.3±1.4	0.3±1.4
GRI	14.2 [7.1, 28.9]	14.6 [7.1, 31.2]
Hypoglycaemia component of GRI	0.2 [0.0, 1.7]	0.1 [0.0, 1.3]
Hyperglycemia component of GRI	6.0 [1.9, 12.4]	6.6 [2.2, 16.1]

Data are mean±SD or n (%), median [interquartile ranges].

HbA1c, haemoglobin A1c; SD, standard deviation; CV, coefficient of variation; TIR, time in range; TAR, time above range; TBR, time below range; LBGI, low blood glucose index;

HBGI, high blood glucose index; MODD, means of daily differences; IQR, interquartile range; GRI, glycemia risk index; IMT, intima media thickness; CCA, common carotid artery; GSM, gray scale median; baPWV, brachial-ankle pulse wave velocity.

Table S2. Clinical characteristics of study participants.

Variables	GRI Q1 (<8.33)	GRI Q2 (≥8.33 to 16.35<)	GRI Q3 (≥16.35 to <31.25)	GRI Q4 (≥31.25)	*p*-value
n	248	245	256	250	
Age, years	63.3±9.1	64.7±9.9	65.8±8.7	64.4±10.7	0.015
Gender, males/females	149/99	146/99	160/96	153/97	0.914†
Duration of diabetes, years	10.4±7.8	12.2±8.1	14.4±8.9	14.3±8.6	<0.001
Body mass index, kg/m^2	24.9±3.7	24.5±3.9	24.7±3.9	24.3±3.9	0.321
Systolic blood pressure, mmHg	130.1±14.8	132.2±14.1	130.7±13.4	132.0±16.8	0.245
Diastolic blood pressure, mmHg	76.0±11.1	76.1±10.5	74.8±11.2	75.3±11.2	0.431
HbA1c, %	6.6±0.5	6.8±0.6	7.1±0.6	7.7±1.1	<0.001
Total cholesterol, mg/dL	188.0±32.3	184.2±29.7	184.6±29.8	186.6±34.3	0.604
LDL cholesterol, mg/dL	105.8±26.3	102.5±25.6	101.2±24.9	103.1±28.9	0.294
HDL cholesterol, mg/dL	60.6±16.1	60.6±15.0	60.5±15.1	59.8±16.5	0.878
Triglycerides, mg/dL	95.5 [69.5, 135.0]	95.0 [70.0, 136.0]	102.0 [73.5, 145.0]	144.0 [74.0, 144.0]	0.181
Uric acid, mg/dL	5.2±1.2	5.2±1.2	5.1±1.2	5.1±1.3	0.554
eGFR, mL/min/1.73 m^2	73.2±17.2	74.9±19.0	73.1±20.0	72.5±25.5	0.235
u-Alb/Cr, mg/g・Cre	10.0 [5.5, 28.3]	12.7 [6.0, 31.2]	14.9 [7.1, 45.2]	24.2 [8.3, 84.7]	<0.001
Neuropathy, n (%)	51 (20.6)	60 (24.5)	65 (25.4)	110 (44.0)	<0.001†
Retinopathy, n (%)	34 (13.7)	49 (20.0)	58 (22.7)	81 (32.4)	<0.001†
Nephropathy, n (%)	39 (15.7)	58 (23.7)	72 (28.1)	101 (40.4)	<0.001†
Use of oral glucose-lowering agents, n (%)					
Metformin	131 (52.8)	141 (57.6)	144 (56.3)	127 (50.8)	0.409†
Sulfonylurea	14 (5.6)	25 (10.2)	39 (15.2)	49 (19.6)	<0.001†
Glinide	8 (3.2)	16 (6.5)	17 (6.6)	27 (10.8)	0.010†
Dipeptidyl peptidase-4 inhibitors	144 (58.1)	138 (56.3)	147 (57.4)	148 (59.2)	0.932†
Sodium-glucose cotransporter-2 inhibitors	48 (19.4)	53 (21.6)	65 (25.4)	65 (26.0)	0.241†
Thiazolidinediones	26 (10.5)	39 (15.9)	37 (14.5)	41 (16.4)	0.224†
α-glucosidase inhibitor	56 (22.6)	46 (18.8)	33 (12.9)	37 (14.8)	0.020†
Glucagon-like peptide-1 antagonists	11 (4.4)	11 (4.5)	21 (8.2)	31 (12.4)	0.001†
Insulin	7 (2.8)	23 (9.4)	48 (18.8)	80 (32.0)	<0.001†
Use of antihypertensive drugs, n (%)	116 (46.8)	116 (47.3)	131 (51.2)	120 (48.0)	0.759†
ACE inhibitors	5 (2.0)	8 (3.3)	7 (2.7)	8 (3.2)	0.824†
Angiotensin II receptor blockers	99 (39.9)	95 (38.8)	101 (39.5)	95 (38.0)	0.974†
Calcium channel blockers	61 (24.6)	67 (27.3)	85 (33.2)	60 (24.0)	0.079†
Use of lipid-lowering agents, n (%)	151 (60.9)	158 (64.8)	144 (56.5)	142 (56.8)	0.194†
Statins	126 (50.8)	134 (54.9)	124 (48.6)	124 (49.6)	0.518†
Ezetimibe	33 (13.3)	33 (13.5)	22 (8.6)	19 (7.6)	0.059†
Fibrates	9 (3.6)	7 (2.9)	11 (4.3)	14 (5.6)	0.469†
Use of antithrombotic agents, n (%)	18 (7.3)	13 (5.3)	17 (6.6)	16 (6.4)	0.845†
Antiplatelet agents	13 (5.2)	11 (4.5)	11 (4.3)	15 (6.0)	0.812†
Anticoagulants	6 (2.4)	2 (0.8)	6 (2.3)	1 (0.4)	0.140†
FLP-CGM-derived metrics					
Mean glucose, mg/dL	119.8±10.3	129.8±12.7	140.7±20.1	171.2±44.9	<0.001
SD, mg/dL	26.1±4.6	33.8±4.9	38.9±7.4	47.9±12.6	<0.001
CV, %	21.8±3.7	26.1±3.9	27.9±4.8	29.0±7.2	<0.001
TIR, %	95.5±2.5	87.8±3.7	78.0±7.0	54.5±19.0	<0.001
TAR$^{>180mg/dL}$, %	3.9±2.7	11.0±5.0	19.7±9.7	40.9±23.9	<0.001
TAR$^{>250mg/dL}$, %	0.1±0.2	0.4±0.4	1.9±1.9	12.9±12.9	<0.001
TBR$^{<70mg/dL}$, %	0.5±0.7	1.2±1.6	2.3±3.3	4.6±8.1	0.101
TBR$^{<54mg/dL}$, %	0.0±0.1	0.1±0.2	0.2±0.5	1.0±2.9	<0.001
GRI	4.6 [2.8, 6.4]	12.1 [10.4, 13.8]	22.2 [18.9, 26.9]	47.0 [36.9, 62.9]	<0.001
Hypoglycaemia component of GRI	0.2 [0.0, 0.6]	0.2 [0.0, 1.7]	0.2 [0.0, 3.2]	0.0 [0.0, 6.4]	0.100
Hyperglycemia component of GRI	1.9 [0.8, 3.0]	6.2 [4.4, 7.7]	11.6 [7.4, 15.4]	24.9 [16.7, 35.1]	<0.001
Ultrasonographic scans of the artery, n	168	161	135	136	
Mean-IMT, mm	0.74±0.17	0.76±0.16	0.77±0.13	0.76±0.13	0.121
CCA-max, mm	1.07±0.42	1.09±0.36	1.16±0.52	1.13±0.47	0.067
Mean-GSM	52.5±20.3	49.0±19.3	48.4±18.6	43.8±17.7	<0.001
Thickened lesion-GSM	48.7±21.4	43.4±19.1	43.0±19.7	37.9±15.6	<0.001
Plaque-GSM	66.1±31.8	62.4±29.4	57.5±24.1	59.0±32.6	0.075
Arterial stiffness, n	124	111	95	115	
PWV, cm/s	1591±305	1700±312	1712±306	1828±474	<0.001

Data are mean±SD or n (%), median [interquartile range].

The data were divided into four groups based on quartiles (Q1-Q4).

p values represent differences among the GRI groups (Q1, Q2, Q3 and Q4), assessed by the Kruskal-Wallis test.

† χ^2 test was used to determine the association between differences among the GRI groups (Q1, Q2, Q3 and Q4).

See Table S1 for abbreviations.

Table S3. Association of GRI with thickened-GSM and plaque-GSM.

		mean-IMT (n=600)		mean-GSM (n=599)	
		β (95% CI)	p value	β (95% CI)	p value
**Model 1**					
GRI		0.0002 (-0.0004, 0.0008)	0.458	-0.1485 (-0.2242, -0.0728)	<0.001
**Model 3**					
GRI		0.0002 (-0.0005, 0.0009)	0.610	-0.1407 (-0.2331, -0.0484)	0.003
Age		0.0045 (0.0030, 0.0060)	<0.001	-0.3610 (-0.5528, -0.1692)	<0.001
Gender, female		-0.0415 (-0.0722, -0.0109)	0.008	-1.8507 (-5.7296, 2.0281)	0.349
BMI		-0.0024 (-0.0059, 0.0012)	0.191	-1.0667 (-1.5171, -0.6163)	<0.001
Duration of diabetes		-0.0001 (-0.0017, 0.0015)	0.911	-0.2963 (-0.5001, -0.0925)	0.004
HbA1c		-0.0114 (-0.0299, 0.0071)	0.226	0.7561 (-1.5790, 3.0913)	0.525
Systolic blood pressure		0.0015 (0.0007, 0.0023)	<0.001	0.0720 (-0.0314, 0.1753)	0.172
LDL cholesterol		0.0100 (-0.0090, 0.0289)	0.302	0.0538 (-2.3424, 2.4500)	0.965
HDL cholesterol		-0.0213 (-0.0540, 0.0114)	0.202	4.3644 (0.2237, 8.5051)	0.039
Log-transformed triglycerides		-0.0067 (-0.0334, 0.0199)	0.619	-3.9152 (-7.2901, -0.5404)	0.023
eGFR		-0.0002 (-0.0010, 0.0005)	0.530	-0.1261 (-0.2238, -0.0283)	0.012
Uric acid		0.0000 (-0.0002, 0.0002)	0.776	0.0055 (-0.0188, 0.0298)	0.655
Log-transformed u-Alb		0.0013 (-0.0072, 0.0098)	0.759	-0.2198 (-1.2985, 0.8589)	0.689
Smoking	Never	Reference	0.169	Reference	0.102
	Current	0.0154 (-0.0193, 0.0502)		4.7904 (0.3978, 9.1830)	
	Former	0.0281 (-0.0011, 0.0574)		1.8181 (-1.8809, 5.5170)	
Alcohol consumption		-0.0235 (-0.0488, 0.0017)	0.067	-0.7622 (-3.9524, 2.4279)	0.639
Use of insulin therapy		-0.0162 (-0.0517, 0.0193)	0.371	-3.1475 (-7.6654, 1.3705)	0.172
Use of ACE-i and/or ARB		0.0083 (-0.0173, 0.0340)	0.524	5.0856 (1.8412, 8.3300)	0.002
Use of statin		0.0131 (-0.0124, 0.0387)	0.314	-5.5566 (-8.7932, -2.3200)	<0.001
Use of antiplatelet agents		0.0165 (-0.0412, 0.0742)	0.575	-5.6896 (-12.9889, 1.6098)	0.126
Presence of retinopathy		0.0215 (-0.0079, 0.0509)	0.152	3.1160 (-0.6072, 6.8391)	0.101

Data are results of univariable and multivariable linear regression analysis.

GRI, gray-scale median; CI, confidence interval; BMI, body mass index; HbA1c, haemoglobin A1c; log-transformed u-Alb, log-transformed urine albumin-to-creatinine ratio; ACE, angiotensin-converting enzyme; HbA1c, haemoglobin A1c; HDL, high-density

lipoprotein; LDL, low-density lipoprotein; eGFR, estimated glomerular filtration rate; ACE-i and/or ARB, ACE inhibitor and/or Angiotensin II receptor blockers.

Table S4. Association of GRI with baPWV.

		mean-baPWV (n=445)	
		β (95% CI)	p value
**Model 1**			
GRI		3.5628 (2.0579, 5.0677)	<0.001
**Model 3**			
GRI		2.9531 (1.1942, 4.7120)	0.001
Age		13.6400 (9.8194, 17.4607)	<0.001
Gender, female		41.8603 (-34.6854, 118.4061)	0.283
BMI		-14.7629 (-23.8005, -5.7253)	0.001
Duration of diabetes		4.6420 (0.8364, 8.4477)	0.017
HbA1c		-63.7979 (-113.1121, -14.4838)	0.011
Systolic blood pressure		6.2756 (4.2782, 8.2731)	<0.001
LDL cholesterol		-32.2136 (-81.4314, 17.0042)	0.199
HDL cholesterol		-34.9423 (-117.1812, 47.2967)	0.404
Log-transformed triglycerides		56.4067 (-6.3806, 119.1940)	0.078
eGFR		0.4226 (-1.3764, 2.2216)	0.645
Uric acid		0.0807 (-0.3801, 0.5415)	0.731
Log-transformed u-Alb		42.5772 (21.7169, 63.4375)	<0.001
Smoking	Never	Reference	0.375
	Current	-33.8316 (-118.5544, 50.8912)	
	Former	23.6535 (-47.6302, 94.9372)	
Alcohol consumption		40.9810 (-21.1981, 103.1600)	0.196
Use of insulin therapy		55.2452 (-33.3559, 143.8462)	0.221
Use of ACE-i and/or ARB		-17.2276 (-80.3899, 45.9346)	0.592
Use of statin		-64.4812 (-126.4853, -2.4770)	0.042
Use of antiplatelet agents		-9.1849 (-152.0688, 133.6991)	0.900
Presence of retinopathy		17.4015 (-53.5905, 88.3934)	0.630

Results of univariable and multivariable linear regression analysis.

See Table S2 for abbreviations.

Table S5. Association of GRI with thickened-GSM and plaque-GSM.

		Thickened-GSM (n=566)		plaque-GSM (n=490)	
		β (95% CI)	p value	β (95% CI)	p value
**Model 1**					
GRI		-0.1469 (-0.2253, -0.0685)	<0.001	-0.1052 (-0.2341, 0.0237)	0.109
**Model 2**					
GRI		-0.0580 (-0.1581, 0.0422)	0.256	0.1214 (-0.0444, 0.2873)	0.151
Age		-0.4038 (-0.6165, -0.1911)	<0.001	-0.2668 (-0.6229, 0.0893)	0.142
Gender, female		4.3803 (0.0524, 8.7082)	0.047	7.6452 (0.4062, 14.8842)	0.039
BMI		-0.0563 (-0.5540, 0.4414)	0.824	-0.8034 (-1.6349, 0.0282)	0.058
Duration of diabetes		0.0042 (-0.2238, 0.2321)	0.971	-0.0891 (-0.4605, 0.2824)	0.638
HbA1c		-0.9404 (-3.4778, 1.5970	0.467	-5.0462 (-9.1789, -0.9134)	0.017
Systolic blood pressure		0.0362 (-0.0766, 0.1490	0.528	0.0921 (-0.0939, 0.2780)	0.331
Total cholesterol		0.2657 (-2.3244, 2.8558)	0.84	-0.9534 (-5.3296, 3.4228)	0.669
HDL cholesterol		3.1665 (-1.9280, 8.2609)	0.223	-1.4696 (-9.9365, 6.9973)	0.733
Log-transformed triglycerides		-1.7999 (-5.6689, 2.0691)	0.361	1.2847 (-5.1388, 7.7081)	0.694
eGFR		-0.0779 (-0.1861, 0.0304)	0.158	-0.0625 (-0.2564, 0.1314)	0.527
Uric acid		-0.0226 (-0.0491, 0.004)	0.095	-0.0123 (-0.0579, 0.0334)	0.598
Log-transformed u-Alb		-0.6262 (-1.802, 0.5496)	0.296	-0.2146 (-2.1723, 1.7431)	0.830
Smoking	Never	Reference	0.215	Reference	0.985
	Current	3.2114 (-1.6750, 8.0979)		0.6148 (-7.600, 8.8296)	
	Former	3.4087 (-0.6595, 7.4768)		0.5170 (-6.2131, 7.2472)	
Alcohol consumption		-1.0273 (-4.5645, 2.5100	0.569	2.5833 (-3.4446, 8.6112)	0.400
Use of insulin therapy		-6.8097 (-11.7207, -1.8988)	0.007	-12.4695 (-20.5113, -4.4277)	0.002
Use of ACE-i and/or ARB		4.8544 (1.2944, 8.4143)	0.008	5.6821 (-0.3544, 11.7186)	0.065
Use of statin		-2.9763 (-6.5741, 0.6215)	0.105	-1.3764 (-7.5155, 4.7627)	0.660
Use of antiplatelet agents		-7.8402 (-15.7264, 0.0460)	0.051	-16.9155 (-29.8871, -3.9440)	0.011
Presence of retinopathy		-1.4548 (-5.5332, 2.6236)	0.484	0.9959 (-5.7649, 7.7567)	0.772

Results of univariable and multivariable linear regression analysis.

See Table S2 for abbreviations.

Table S6. Association of the hypoglycaemia component of GRI with intima-media thickness and gray-scale median.

		mean-IMT (n=600)		mean-GSM (n=599)	
		β (95% CI)	p value	β (95% CI)	p value
**Model 1**					
GRI		0.0029 (-0.0002, 0.0060)	0.063	0.0665 (-0.3209, 0.4538)	0.736
**Model 2**					
GRI		0.0010 (-0.0021, 0.0041)	0.534	-0.2125 (-0.6080, 0.1831)	0.292
Age		0.0045 (0.0030, 0.0061)	<0.001	-0.3604 (-0.5573, -0.1635)	<0.001
Gender, female		-0.0364 (-0.0674, -0.0053)	0.022	-1.3820 (-5.3409, 2.5768)	0.493
BMI		-0.0023 (-0.0059, 0.0013)	0.216	-1.0456 (-1.5099, -0.5812)	<0.001
Duration of diabetes		-0.0002 (-0.0018, 0.0015)	0.850	-0.3062 (-0.5191, -0.0932)	0.005
HbA1c		-0.0055 (-0.0219, 0.0108)	0.507	-1.2531 (-3.3349, 0.8287)	0.238
Systolic blood pressure		0.0015 (0.0007, 0.0023)	<0.001	0.0810 (-0.0247, 0.1866)	0.133
Total cholesterol		0.0052 (-0.0136, 0.0241)	0.585	0.0098 (-2.3988, 2.4185)	0.994
HDL cholesterol		-0.0244 (-0.0617, 0.0129)	0.200	3.2653 (-1.4897, 8.0203)	0.178
Log-transformed triglycerides		-0.0073 (-0.0358, 0.0213)	0.618	-4.3044 (-7.9472, -0.6617)	0.021
eGFR		-0.0003 (-0.0011, 0.0005)	0.521	-0.1197 (-0.2210, -0.0183)	0.021
Uric acid		0.0000 (-0.0002, 0.0002)	0.735	0.0077 (-0.0171, 0.0325)	0.542
Log-transformed u-Alb		0.0015 (-0.0072, 0.0102)	0.732	-0.3542 (-1.4615, 0.7531)	0.530
Smoking	Never	Reference	0.190	Reference	0.199
	Current	0.0182 (-0.0170, 0.0534)		4.0999 (-0.3879, 8.5877)	
	Former	0.0275 (-0.0023, 0.0573)		1.8006 (-1.9962, 5.5973)	
Alcohol consumption		-0.0206 (-0.0463, 0.0051)	0.116	-0.4647 (-3.7389, 2.8095)	0.781
Use of insulin therapy		-0.0142 (-0.0502, 0.0219)	0.441	-4.8444 (-9.4635, -0.2254)	0.040
Use of ACE-i and/or ARB		0.0078 (-0.0185, 0.0341)	0.561	5.3804 (2.0242, 8.7366)	0.002
Use of statin		0.0084 (-0.0179, 0.0347)	0.533	-5.3414 (-8.6945, -1.9883)	0.002
Use of antiplatelet agents		0.0175 (-0.0417, 0.0766)	0.562	-5.7240 (-13.2627, 1.8148)	0.136
Presence of retinopathy		0.0200 (-0.0101, 0.0501)	0.192	3.3069 (-0.5286, 7.1425)	0.091

Results of univariable and multivariable linear regression analysis.

See Table S2 for abbreviations.

Table S7. Association of the hyperglycaemia component of GRI with intima-media thickness and gray-scale median.

		mean-IMT (n=600)		mean-GSM (n=599)	
		β (95% CI)	p value	β (95% CI)	p value
**Model 1**					
GRI		-0.0003 (-0.0012, 0.0007)	0.582	-0.2565 (-0.3777, -0.1352)	<0.001
**Model 2**					
GRI		0.0002 (-0.0012, 0.0017)	0.759	-0.2641 (-0.4510, -0.0772)	0.006
Age		0.0045 (0.0030, 0.0061)	<0.001	-0.3319 (-0.5283, -0.1354)	<0.001
Gender, female		-0.0353 (-0.0667, -0.0039)	0.028	-2.3156 (-6.2941, 1.6628)	0.253
BMI		-0.0023 (-0.0060, 0.0013)	0.211	-1.0592 (-1.5207, -0.5978)	<0.001
Duration of diabetes		-0.0001 (-0.0018, 0.0016)	0.902	-0.3404 (-0.5525, -0.1282)	0.002
HbA1c		-0.0096 (-0.0331, 0.0139)	0.424	2.1476 (-0.8285, 5.1238)	0.157
Systolic blood pressure		0.0015 (0.0007, 0.0023)	<0.001	0.0696 (-0.0353, 0.1745)	0.193
Total cholesterol		0.0050 (-0.0139, 0.0239)	0.604	0.1834 (-2.2125, 2.5793)	0.881
HDL cholesterol		-0.0246 (-0.0620, 0.0129)	0.198	3.8037 (-0.9404, 8.5478)	0.116
Log-transformed triglycerides		-0.0076 (-0.0362, 0.0210)	0.600	-4.1082 (-7.7289, -0.4876)	0.026
eGFR		-0.0003 (-0.0011, 0.0005)	0.463	-0.1118 (-0.2116, -0.0119)	0.028
Uric acid		0.0000 (-0.0002, 0.0002)	0.735	0.0070 (-0.0177, 0.0316)	0.580
Log-transformed u-Alb		0.0015 (-0.0072, 0.0102)	0.736	-0.2652 (-1.3680, 0.8375)	0.637
Smoking	Never	Reference	0.190	Reference	0.188
	Current	0.0185 (-0.0167, 0.0537)		4.1549 (-0.3033, 8.6130)	
	Former	0.0281 (-0.0017, 0.0579)		1.5606 (-2.2094, 5.3305)	
Alcohol consumption		-0.0206 (-0.0462, 0.0051)	0.117	-0.5567 (-3.8118, 2.6985)	0.737
Use of insulin therapy		-0.0121 (-0.0474, 0.0233)	0.502	-5.0204 (-9.5205, -0.5203)	0.029
Use of ACE-i and/or ARB		0.0085 (-0.0178, 0.0348)	0.528	5.1124 (1.7812, 8.4435)	0.003
Use of statin		0.0077 (-0.0185, 0.0339)	0.565	-5.2540 (-8.5737, -1.9344)	0.002
Use of antiplatelet agents		0.0168 (-0.0425, 0.0761)	0.577	-5.0608 (-12.5680, 2.4464)	0.186
Presence of retinopathy		0.0200 (-0.0101, 0.0501)	0.193	3.0869 (-0.7289, 6.9027)	0.113

Results of univariable and multivariable linear regression analysis.

See Table S2 for abbreviations.

Table S8. Association of the hypoglycaemia component of GRI with thickened-GSM and plaque-GSM.

		Thickened-GSM (n=566)		plaque-GSM (n=490)	
		β (95% CI)	p value	β (95% CI)	p value
**Model 1**					
GRI		0.0718 (-0.3309, 0.4746)	0.726	0.8433 (0.2071, 1.4794)	0.009
**Model 2**					
GRI		0.0418 (-0.3801, 0.4638)	0.846	0.9523 (0.2620, 1.6425)	0.007
Age		-0.4115 (-0.6240, -0.1989)	<0.001	-0.2405 (-0.5947, 0.1138)	0.183
Gender, female		4.5878 (0.2673, 8.9083)	0.037	6.8714 (-0.3092, 14.0519)	0.061
BMI		-0.0389 (-0.5370, 0.4592)	0.878	-0.7808 (-1.6073, 0.0457)	0.064
Duration of diabetes		0.0050 (-0.2236, 0.2336)	0.966	-0.1316 (-0.5015, 0.2384)	0.485
HbA1c		-1.6695 (-3.8896, 0.5506)	0.140	-2.0077 (-5.6389, 1.6235)	0.278
Systolic blood pressure		0.0341 (-0.0792, 0.1473)	0.555	0.0710 (-0.1147, 0.2567)	0.453
Total cholesterol		0.2394 (-2.3540, 2.8329)	0.856	-0.7757 (-5.1276, 3.5762)	0.726
HDL cholesterol		2.9354 (-2.1525, 8.0234)	0.258	-1.0115 (-9.4110, 7.3879)	0.813
Log-transformed triglycerides		-1.7984 (-5.6729, 2.0760)	0.362	1.5439 (-4.8439, 7.9316)	0.635
eGFR		-0.0720 (-0.1811, 0.0372)	0.196	-0.0317 (-0.2262, 0.1627)	0.749
Uric acid		-0.0223 (-0.0489, 0.0042)	0.099	-0.0105 (-0.0558, 0.0349)	0.650
Log-transformed u-Alb		-0.6706 (-1.8458, 0.5046)	0.263	-0.1726 (-2.1172, 1.7719)	0.862
Smoking	Never	Reference	0.231	Reference	0.982
	Current	3.0723 (-1.8191, 7.9637)		0.7473 (-7.4159, 8.9104)	
	Former	3.3656 (-0.7127, 7.4438)		0.0735 (-6.6244, 6.7714)	
Alcohol consumption		-0.9683 (-4.5084, 2.5718)	0.591	2.4344 (-3.5568, 8.4257)	0.425
Use of insulin therapy		-7.4228 (-12.3610, -2.4846)	0.003	-13.4889 (-21.4878, -5.4899)	<0.001
Use of ACE-i and/or ARB		4.7864 (1.2168, 8.3559)	0.009	5.2369 (-0.7755, 11.2494)	0.088
Use of statin		-2.8091 (-6.4178, 0.7996)	0.127	-0.8915 (-7.0037, 5.2207)	0.774
Use of antiplatelet agents		-8.0619 (-15.9484, -0.1753)	0.045	-16.5759 (-29.4388, -3.7129)	0.012
Presence of retinopathy		-1.3100 (-5.3897, 2.7697)	0.528	0.9094 (-5.8006, 7.6193)	0.790

Results of univariable and multivariable linear regression analysis.

See Table S2 for abbreviations.

Table S9. Association of the hyperglycaemia component of GRI with thickened-GSM and plaque-GSM.

		Thickened-GSM (n=566)		plaque-GSM (n=490)	
		β (95% CI)	p value	β (95% CI)	p value
**Model 1**					
GRI		-0.2511 (-0.3766, -0.1256)	<0.001	-0.3400 (-0.5432, -0.1368)	0.001
**Model 2**					
GRI		-0.1440 (-0.3444, 0.0563)	0.158	-0.0899 (-0.4304, 0.2506)	0.604
Age		-0.3967 (-0.6099, -0.1836)	<0.001	-0.2565 (-0.6140, 0.1010)	0.159
Gender, female		4.1022 (-0.2620, 8.4664)	0.065	6.9837 (-0.3184, 14.2859)	0.061
BMI		-0.0560 (-0.5531, 0.4411)	0.825	-0.8399 (-1.6727, -0.0071)	0.048
Duration of diabetes		-0.0097 (-0.2385, 0.2192)	0.934	-0.1086 (-0.4834, 0.2662)	0.569
HbA1c		-0.0138 (-3.2169, 3.1894)	0.993	-2.3510 (-7.7600, 3.0580)	0.393
Systolic blood pressure		0.0308 (-0.0820, 0.1437)	0.592	0.0977 (-0.0885, 0.2839)	0.303
Total cholesterol		0.3101 (-2.2798, 2.9000)	0.814	-0.9283 (-5.3145, 3.4579)	0.678
HDL cholesterol		3.3529 (-1.7553, 8.4610)	0.198	-0.7859 (-9.3248, 7.7529)	0.857
Log-transformed triglycerides		-1.7126 (-5.5811, 2.1558)	0.385	1.3578 (-5.0845, 7.8000)	0.679
eGFR		-0.0730 (-0.1809, 0.0349)	0.184	-0.0807 (-0.2736, 0.1121)	0.411
Uric acid		-0.0226 (-0.0491, 0.0039)	0.095	-0.0124 (-0.0581, 0.0333)	0.595
Log-transformed u-Alb		-0.6021 (-1.7784, 0.5743)	0.315	-0.1241 (-2.0888, 1.8406)	0.901
Smoking	Never	Reference	0.235	Reference	0.979
	Current	3.1849 (-1.6955, 8.0653)		0.8491 (-7.3868, 9.0850)	
	Former	3.2677 (-0.8008, 7.3362)		0.4357 (-6.3171, 7.1885)	
Alcohol consumption		-1.0508 (-4.5860, 2.4844)	0.560	2.4699 (-3.5716, 8.5114)	0.422
Use of insulin therapy		-7.1403 (-11.9737, -2.3069)	0.004	-11.1881 (-19.1204, -3.2559)	0.006
Use of ACE-i and/or ARB		4.7511 (1.1936, 8.3085)	0.009	5.8209 (-0.2246, 11.8665)	0.059
Use of statin		-2.8789 (-6.4670, 0.7091)	0.116	-1.8191 (-7.9501, 4.3119)	0.560
Use of antiplatelet agents		-7.7254 (-15.6107, 0.1600)	0.055	-15.9955 (-28.9914, -2.9995)	0.016
Presence of retinopathy		-1.5125 (-5.5903, 2.5652)	0.467	0.5450 (-6.2427, 7.3327)	0.875

Results of univariable and multivariable linear regression analysis.

See Table S2 for abbreviations.

Table S10. Association of the hypoglycaemia component of GRI with brachial-ankle pulse wave velocity (baPWV).

		mean-baPWV (n=445)	
		β (95% CI)	p value
**Model 1**			
GRI		19.1962 (9.9577, 28.4348)	<0.001
**Model 2**			
GRI		9.8682 (1.5155, 18.2208)	0.021
Age		14.7396 (10.8640, 18.6152)	<0.001
Gender, female		43.1531 (-33.8389, 120.1451)	0.271
BMI		-14.6358 (-23.7923, -5.4792)	0.002
Duration of diabetes		3.7681 (-0.1461, 7.6822)	0.059
HbA1c		-4.6154 (-45.6365, 36.4058)	0.825
Systolic blood pressure		6.2779 (4.2595, 8.2963)	<0.001
Total cholesterol		-41.8849 (-89.9351, 6.1653)	0.087
HDL cholesterol		11.4704 (-80.4192, 103.3599)	0.806
Log-transformed triglycerides		86.3694 (19.6126, 153.1262)	0.011
eGFR		0.7247 (-1.1305, 2.5800)	0.443
Uric acid		0.0424 (-0.4245, 0.5093)	0.858
Log-transformed u-Alb		46.5649 (25.4736, 67.6562)	<0.001
Smoking	Never	Reference	0.546
	Current	-5.4955 (-90.2715, 79.2806)	
	Former	33.1339 (-39.0676, 105.3354)	
Alcohol consumption		35.8093 (-27.5669, 99.1855)	0.267
Use of insulin therapy		61.5405 (-29.2004, 152.2813)	0.183
Use of ACE-i and/or ARB		-15.4494 (-80.1859, 49.2870)	0.639
Use of statin		-72.9231 (-136.5835, -9.2628)	0.025
Use of antiplatelet agents		36.1783 (-105.2809, 177.6375)	0.615
Presence of retinopathy		14.0044 (-58.3683, 86.3771)	0.704

Results of univariable and multivariable linear regression analysis.

See Table S2 for abbreviations.

Table S11. Association of the hyperglycaemia component of GRI with brachial-ankle pulse wave velocity (baPWV).

		mean-baPWV (n=445)	
		β (95% CI)	p value
**Model 1**			
GRI		3.5782 (1.1697, 5.9868)	0.004
**Model 2**			
GRI		4.6723 (1.4361, 7.9085)	0.005
Age		14.0063 (10.1116, 17.9010)	<0.001
Gender, female		58.7452 (-18.1614, 135.6519)	0.134
BMI		-14.2335 (-23.3655, -5.1016)	0.002
Duration of diabetes		4.5344 (0.6516, 8.4172)	0.022
HbA1c		-74.6522 (-132.3181, -16.9864)	0.011
Systolic blood pressure		6.4006 (4.3883, 8.4129)	<0.001
Total cholesterol		-41.2593 (-89.1157, 6.5972)	0.091
HDL cholesterol		3.4843 (-88.4119, 95.3805)	0.941
Log-transformed triglycerides		70.1944 (3.2340, 137.1547)	0.040
eGFR		0.1690 (-1.6658, 2.0038)	0.856
Uric acid		0.0887 (-0.3778, 0.5551)	0.709
Log-transformed u-Alb		43.2046 (21.9887, 64.4206)	<0.001
Smoking	Never	Reference	0.376
	Current	-18.2063 (-103.4207, 67.0082)	
	Former	35.8374 (-36.0682, 107.7431)	
Alcohol consumption		42.7492 (-20.3884, 105.8868)	0.184
Use of insulin therapy		75.6634 (-13.0698, 164.3966)	0.094
Use of ACE-i and/or ARB		-10.5863 (-74.9832, 53.8107)	0.747
Use of statin		-73.0460 (-136.4806, -9.6114)	0.024
Use of antiplatelet agents		-0.0011 (-141.0350, 141.0329)	1.000
Presence of retinopathy		15.3175 (-56.8332, 87.4681)	0.677

Results of univariable and multivariable linear regression analysis.

See Table S2 for abbreviations.

Supplementary Material

Alphabetically arranged list of medical facilities and investigators.

Ashiya Central Hospital: Koji Matsushita, Manabu Narisawa
Aso Clinic: Katsumi Aso, Yuko Ando, Fumihiko Sato
Hagiwara Central Hospital: Emiko Morita, Keiichi Torimoto
Hayashi Clinic: Isao Hayashi
Inokuchi Clinic: Nobuo Inokuchi
Japan Community Health Care Organization Osaka Hospital: Masahiro Hatazaki, Arichika Deguchi, Azusa Shiraki
Juntendo Tokyo Koto Geriatric Medical Center (Department of Medicine, Diabetology and Endocrinology): Ayako Kitamura, Eri Tanabe, Hidenori Yoshii, Tomio Onuma, Tomo Nakajima
Juntendo University Graduate School of Medicine (Department of Metabolism & Endocrinology): Eisuke Yasunari, Hideyoshi Kaga, Hiroaki Sato, Hirotaka Watada, Kagemi Takeno, Luka Suzuki, Miwa Himuro, Syuhei Aoyama, Takashi Funayama, Takehiro Katahira, Takeshi Miyatsuka, Tomoya Mita, Yuya Nishida
Juntendo University Nerima Hospital (Department of Medicine, Diabetes and Endocrinology): Koji Komiya
Kanda Naika Clinic: Satoshi Kawashima
Kansai Rosai Hospital (Diabetes and Endocrinology): Tsunehiko Yamamoto
Kihara Diabetes Clinic: Yasuyuki Kihara
Kosugi Medical Clinic: Keisuke Kosugi
Kawasaki Hospital (Department of Internal Medicine): Akihito Otsuka, Jun Murai
Matsuoka Medical Clinic: Hirofumi Matsuoka
Misaki Naika Clinic: Nobuichi Kuribayashi
Japan Labour Health and Safety Organization Kyushu Rosai Hospital, Moji Medical Center (Department of Internal Medicine): Tadashi Arao, Kei Sugai
Musashino Family Clinic: Yuichi Kojima
Nakakinen Clinic: Maiko Nakata, Miyoko Saito, Takeshi Osonoi, Yusuke Osonoi
Nakama Municipal hospital: Kohei Uriu, Yosifumi Inada, Kanako Suzuka, Ichiro Takagi
National Hospital Organization Osaka National Hospital (Diabetes Center): Ken Kato
Nishida Keiko Diabetes Clinic: Keiko Nishida, Akira Kurozumi, Fumi Uemura, Keiichi Torimoto, Maiko Hajime, Manabu Narisawa, Satomi Sonoda, Kumiko Tidiwa
Osaka General Medical Center (Department of Diabetes and Endocrinology): Youhei Fujita, Sayoko Shimizu, Masahisa Hata, Yutaka Umayahara
Osaka Police Hospital (Department of Endocrinology and Metabolism): Tetsuyuki Yasuda
Osaka Rosai Hospital: Ryomoto Kayoko
Osaka University Graduate School of Medicine (Department of Metabolic Medicine): Iichiro Shimomura, Naoto Katakami, Takaaki Matasuoka, Mitsuyoshi Takahara, Kazuyuki Miyashita, Hiroyo Ninomiya, Naohiro Taya
Sasaki Hospital: Shinichiro Mine, Kenji Koikawa
School of Medicine, University of Occupational and Environmental Health, Japan (First Department of Internal Medicine): Yosuke Okada, Akira Kurozumi, Manabu Narisawa, Maiko Hajime, Fumi Uemura, Satomi Sonoda, Kenichi Tanaka, Takashi Otsuka
Secomedic Hospital: Satomi Wakasugi, Tomoya Mita
Shiraiwa Medical Clinic: Toshihiko Shiraiwa
Taneda Clinic: Yoshinobu Taneda
Takaishi Naika Ichoka Clinic: Tomoya Mita
Tobata General Hospital (Department of Internal Medicine): Kazuko Kanda
Wakamatsu Hospital of the University of Occupational and Environmental Health: Torimoto Keiichi, Nishio Kousuke

Supplemental Fig.1

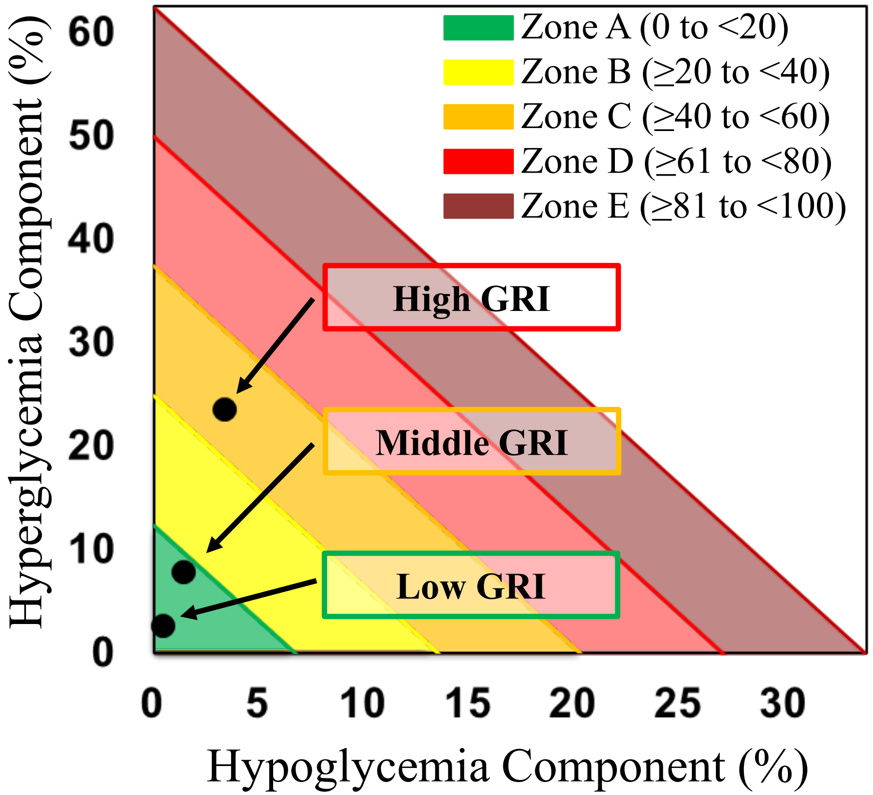

Supplemental Fig.2

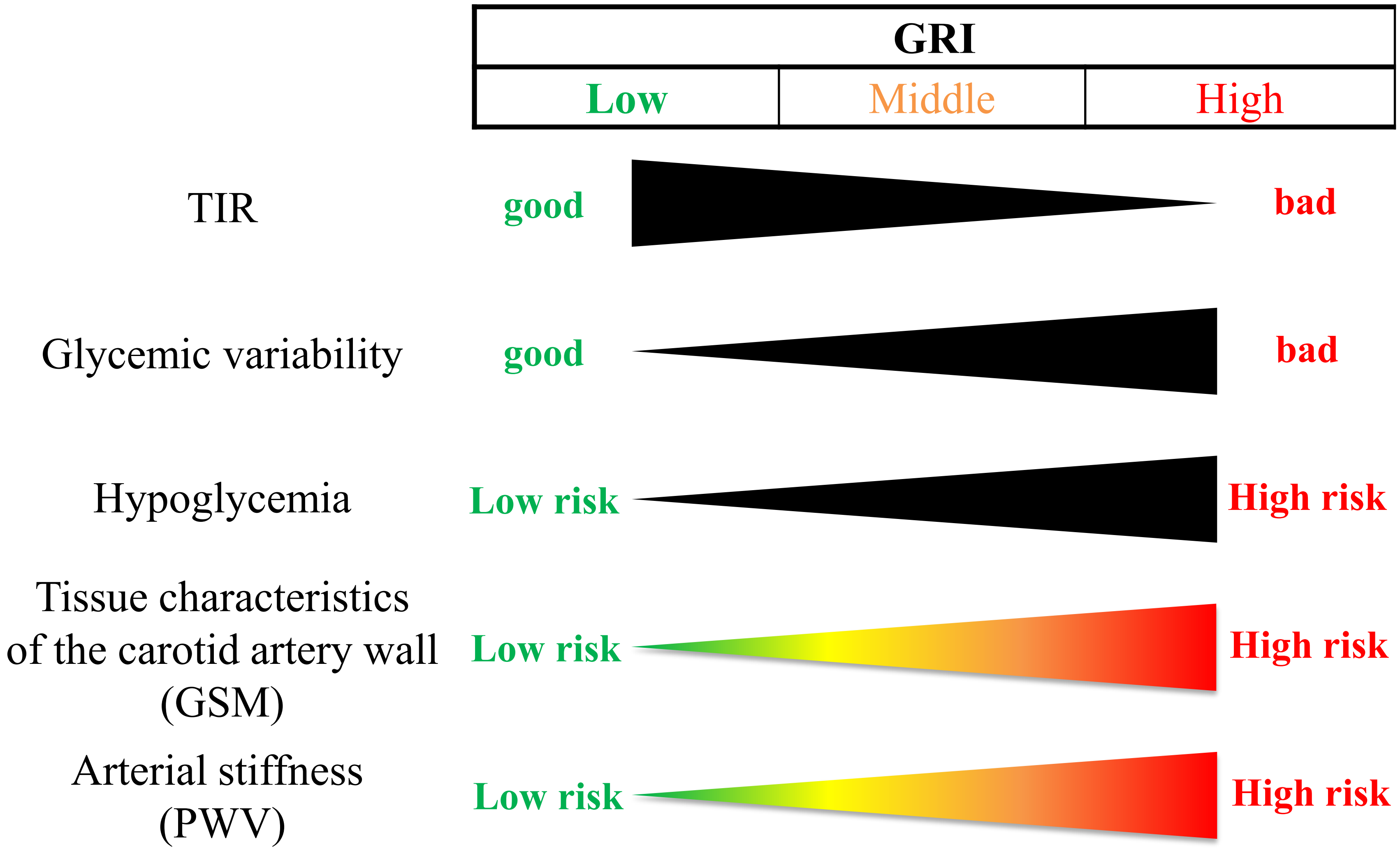

Supplement: Supplementary file 1 — Data S1. Supporting Information. Figure S1. The Glycemia risk index grid. The Glycemia risk index (GRI) is displayed on a two‐dimensional diagram called the GRI grid, which displays the hypocomponent on the abscissa and the hypercomponent on the ordinate. The distance from the origin represents the overall GRI score, which is divided into five zones (A–E), from 0 to 100. These zones are color‐coded on the GRI grid in increments of 20, allowing for a visual evaluation of blood glucose quality. GRI is analyzed in tertiles (Q1–Q3) as follows: Q1 (Low GRI, < 11.15), Q2 (Middle GRI, ≥ 11.15 to < 25.39), and Q3 (High GRI, ≥ 25.39). Figure S2. Summary of this study. This study demonstrates a close association between GRI and ultrasound indices of carotid artery wall tissue characteristics (GSM) and atherosclerosis, as assessed by PWV, in patients with T2DM free of history of cardiovascular events. GRI, glycaemia risk index; GSM, grayscale median; PWV, pulse wave velocity; TRI, Time in range. [file JDB-17-e70065-s001.pdf]
